# A PLUM Job: Peptide modeLs for Understanding and engineering antiMicrobial therapeutics

**DOI:** 10.64898/2026.02.21.707214

**Published:** 2026-02-23

**Authors:** Priyanka Banerjee, Iddo Friedberg, Britta Rued, Oliver Eulenstein

**Affiliations:** 1 Department of Computer Science, Iowa State University, Ames, 50011, IA, USA; 2 Department of Veterinary Microbiology and Preventive Medicine, Iowa State University, Ames, 50011, IA, USA

**Keywords:** Generative Model, Variational Autoencoders, Antimicrobial Peptides, Disentangled Representations, Animal Health, Human Health

## Abstract

**Motivation:**

Antibiotic-resistant infections in humans and animals are rising, creating an urgent need for new antimicrobial strategies. This challenge extends beyond clinical settings to food production systems; the Centers for Disease Control and Prevention estimates that foodborne pathogens cause over 48 million illnesses annually in the U.S. alone. Antimicrobial peptides (AMPs) are a promising alternative, with broad activity and lower risk of resistance. However, rational design remains challenging, especially when simultaneously controlling sequence, function, and peptide length.

**Results:**

We introduce *Peptide modeLs for Understanding and engineering antiMicrobial therapeutics* (PLUM), a structured conditional Variational Autoencoder for controlled AMP generation. PLUM disentangles sequence, function, and length in its latent space, enabling *de novo* and prototype-conditioned generation of peptides 5–35 amino acids long, allowing capture of larger functional domains. Across 45,000 generated peptides, PLUM achieved the highest AMP yield (0.885, 7% higher than HydrAMP) and increased AMP diversity (14% higher than HydrAMP), while maintaining the highest non-AMP sequence yield 0.895 (19% higher than HydrAMP). For prototype-conditioned generation, PLUM produced 37% more AMPs than HydrAMP, generating sequences that closely matched real peptide compositions with low predicted toxicity. Integrated AMP classifiers enabled robust evaluation of identity and potency across diverse bacteria. These results establish PLUM as a scalable, versatile platform for designing AMPs and next-generation therapeutics.

**Availability:**

https://github.com/priyamayur/PLUM

## Introduction

Antimicrobial peptides (AMPs) are naturally occurring small biomolecules, typically consisting of 10–50 amino acids, that serve as the first line of defense against invading pathogens. They are widely distributed in all domains of life and have been extensively studied for their broad-spectrum antimicrobial activities [16, 41]. The discoveries of cecropins in 1981 and magainins in 1987 marked some of the earliest breakthroughs in AMP research [41]. In recent decades, the widespread overuse of conventional antibiotics and the resulting escalation of antibiotic resistance have renewed interest in AMPs as potential therapeutic alternatives. Due to their broad activity spectrum, thermal stability, and low toxicity, AMPs represent a promising class of candidates for next-generation antimicrobial agents [40] for use in humans and animals.

Besides their use as antimicrobial therapeutics, AMPs have also garnered recent interest as a novel method for reducing infections with problematic bacterial species, particularly in livestock and healthcare settings. Current methods in these contexts rely on environmental controls such as surface decontamination and identification of contaminated sources [23, 13]. While these strategies have been successful in reducing the incidence of related outbreaks, they do not eliminate their occurrence. These strategies are also complicated by the systemic overuse of antibiotics. As such, agencies in the United States and the European Union have begun to heavily monitor or have outright banned the application of specific antibiotics in livestock, particularly due to the increase in antimicrobial resistance [1]. As a result, the need for alternative strategies and methods to control bacterial infections has increased. Initial research has demonstrated that AMPs are a viable alternative to modulate pathogen colonization andprovide protective immunological benefits [27, 24]. The use of AMPs provides a novel strategy for controlling this in humans and animals. The goal of designing *Peptide modeLs for Understanding and engineering antiMicrobial therapeutics* (PLUM) was to enable controllable, large-scale generation of novel, easily synthesized AMPs for these applications, representing a departure from the typical use of current models.

Traditionally, the discovery of novel antimicrobial peptides has relied on the experimental modification of existing peptides in laboratory settings. This has been further complicated by the structural complexity that accompanies many of these AMPs [3, 24]. This approach is often time-consuming and constrained in its capacity to explore a diverse range of peptide sequences [30, 8]. These limitations have underscored the need for efficient computational strategies that can accelerate AMP discovery and facilitate the generation of novel, diverse candidates.

Generative models have gained attention for learning complex data distributions and creating realistic samples in images, language, and audio. This has inspired their use in peptide design, enabling AMP generation. Early ”machine learning” research on AMPs primarily focused on sequence classification using traditional algorithms [39, 2, 14, 28, 33]. With deep learning, attention shifted toward generative modeling for designing novel peptides with desired properties. Long short-term memory networks (LSTMs) were among the first models applied to peptide sequence generation [22, 20, 35], followed by VAEs, generative adversarial networks (GANs), Transformers, and diffusion models, driving a surge in AI-guided peptide design. VAEs and GANs were early generative approaches [31, 11, 30, 8]. PepGAN generated both AMPs and non-AMPs, while AMP-GAN introduced conditional control. Conditional VAEs (cVAEs) enabled property-guided generation [8, 9, 30]; PepCVAE followed standard cVAE frameworks [8], and HydrAMP improved diversity through latent space disentanglement [30]. More recently, diffusion models have provided highly controllable generation [37, 4], with ProT-Diff leveraging ProtT5-guided diffusion and TG-CDDPM generating AMPs from text prompts [37, 4].

Many existing models have demonstrated success in de novo AMP generation. However, most focus exclusively on generating AMPs and lack the ability to modulate functional attributes, produce non-AMP sequences, or generate analogues of known peptides. While AMP generation is important, practical applications—such as exploring diverse peptide space or producing sequences with controllable properties—require models that enable flexible and targeted peptide design. HydrAMP is one of the few models capable of generating both AMPs and non-AMPs and, to our knowledge, the only model to explore prototype-conditioned analogue generation. Although it provides a valuable starting point for comprehensive peptide design, HydrAMP conditions sequence generation on *E. coli* Minimum Inhibitory Concentrations (MIC), which may bias the output toward a single species, and it is limited to producing peptides of up to 25 amino acids.

PLUM is a structured conditional VAE model for peptide design, inspired by advances in disentangled representation learning [5, 10, 19, 6] and cVAEs such as Conditional Subspace VAE (CSVAE) [15]. While standard cVAEs often produce entangled latent spaces, explicitly separating label-correlated information into dedicated subspaces facilitates interpretability and targeted manipulation. PLUM extends this principle to peptide sequences by decomposing them into latent subspaces corresponding to general sequence patterns, functional activity, and length. This structured design allows independent control of biologically meaningful properties during generation while maintaining flexibility and diversity. Building on this architecture, PLUM introduces capabilities that address key limitations of existing models, offering four major contributions:

1. Structured, disentangled latent architecture: PLUM’s latent space is explicitly partitioned for sequence, function, and length, supporting interpretable and targeted manipulation of peptide properties.
2. *De novo* generation across functional classes and lengths: PLUM generates both AMPs and non-AMPs, producing sequences from 5 up to 35 amino acids, allowing the model to capture larger functional domains and enabling exploration of diverse peptide space.
3. Prototype-conditioned generation: The model can create peptide analogues of a given sequence, with controllable functional activity and length.
4. Pre-trained classifiers for AMP identity and potency: PLUM includes classifiers that predict AMP vs. non-AMP and estimate antimicrobial potency, allowing quantitative evaluation of generated sequences.

Together, these features establish PLUM as a flexible, interpretable platform for controlled exploration and design of peptide sequences beyond conventional AMP-focused approaches.

## Methods

This section describes the datasets, predictive classifiers, and the PLUM generative framework. First, we curated high-quality datasets of AMPs and non-AMPs, along with associated activity (MIC) data, to provide a reliable ground truth for both classification and generation tasks. Next, we developed two classifiers to assess AMP identity and antimicrobial potency, enabling systematic evaluation of PLUM-generated sequences. Finally, we present the PLUM generative model, which learns disentangled latent representations of peptide sequences to allow controlled and interpretable design of novel AMPs. Together, these components form a comprehensive methodological pipeline for data-driven peptide generation and evaluation.

### Dataset Construction

PLUM was developed and evaluated using curated datasets of natural linear AMPs (5–35 residues) and matched non-AMPs, along with associated activity data (MICs). AMP sequences were collected from CAMP, DRAMP, DBAASP, and GRAMPA [12, 18, 25, 38], filtered for linearity, canonical amino acids, and sequence validity, yielding 4,723 unique sequences. The non-AMP dataset was retrieved from UniProt and filtered similarly, producing a size- and length-matched set of 4,853 peptides. AMP and non-AMP sequences were split using a length-aware, embedding-dissimilar strategy to minimize similarity-driven leakage. The resulting AMP training set contained 8,235 sequences (4,202 positive, 4,033 negative), and the AMP test set contained 1,042 sequences (521 positive, 521 negative).

We also developed the AMP MIC dataset where MIC values were averaged across tested organisms and used to label peptides as active (≤ 10 *μ*M; 1,535 sequences) or inactive (> 10 *μ*M; 1,230 sequences). These datasets provide the training and test data for the classifiers and generative model described below. Full details on data sources, filtering steps, MIC processing, and dataset splits are provided in Supplementary Materials Section 1.1.

### Classification Models

We developed two classification models, for evaluation of PLUM generated peptides:

#### AMP Classifier

This classifier predicts the probability that a peptide is an AMP. It was trained on the AMP training dataset and evaluated on the corresponding test set.

#### AMP Potency Classifier

This classifier predicts whether a peptide is highly active or inactive, based on its average MIC across target organisms. Labels were derived from the AMP MIC dataset (see Dataset Construction), with 75% of peptides used for training and 25% for testing.

#### Sequence Representation and Learning Algorithms

Peptides were represented using pre-trained protein language model embeddings, such as ProtT5 and ESM2, which capture sequence-level and structural patterns. These embeddings served as input features for Support Vector Machine (SVM), Random Forest (RF), and Multilayer Perceptron (MLP) classifiers, allowing us to compare performance across different learning paradigms.

### PLUM Model Framework

PLUM is a deep generative framework for designing novel AMP sequences. Inspired by conditional and structured VAEs [15] and disentangled latent representations in sequential data [5, 10, 19], PLUM encodes peptides into three disentangled latent subspaces. The first latent, $Z_{\text{seq}}$, captures only sequence information, independent of AMP activity or length. The second latent, $Z_{\text{func}}$, models antimicrobial functionality, allowing explicit control over predicted activity. The third latent, $Z_{\text{length}}$, captures the length of the peptide, enabling control of peptide size. By separating these factors, PLUM supports interpretable and controllable generation, as each latent can be independently manipulated during sequence design.

The architecture comprises three main components: an LSTM-based encoder mapping sequences to structured latents, disentangled latent subspaces modeling sequence, function, and length, and an autoregressive decoder reconstructing sequences from these latents.

#### Encoding Disentangled Latent Spaces

PLUM maps a peptide sequence x into three disentangled latent subspaces, $Z_{\text{seq}}$ (sequence patterns), $Z_{\text{func}}$ (antimicrobial activity), and $Z_{\text{length}}$ (length bin $\ell_{\text{bin}}$).

Each latent is an independent multivariate Gaussian via an LSTM encoder:

(1) $$q_{\phi }\left(Z_{\text{seq}}|x\right)=𝒩(Z_{\text{seq}};\mu_{\text{seq}},diag(\sigma_{\text{seq}}^{2})),$$

(2) $$q_{\phi }\left(Z_{\text{func}}|x,y\right)=𝒩(Z_{\text{func}};\mu_{\text{func}},diag(\sigma_{\text{func}}^{2})),$$

(3) $$q_{\phi }\left(Z_{\text{length}}|x,\ell_{\text{bin}}\right)=𝒩(Z_{\text{length}};\mu_{\text{length}},diag(\sigma_{\text{length}}^{2})),$$

with means $\mu_{k}$ and variances $\sigma_{k}^{2}$ predicted by the encoder (log-variances for stability).

Latents are sampled via the reparameterization trick, $Z_{k}=\mu_{k}+\sigma_{k}⊙\epsilon_{k}$, $\epsilon_{k}∼𝒩(0,I)$. This structure allows independent control of each latent for peptide generation

#### Decoder

The PLUM decoder reconstructs peptide sequences from the disentangled latent subspaces $Z_{\text{seq}}$, $Z_{\text{func}}$, and $Z_{\text{length}}$ using an autoregressive LSTM. Each amino acid is predicted conditioned on previously generated residues and the latent codes.

For a sequence of length L, the generative distribution factorizes as

(4) $$p_{\theta }\left(x|Z_{\text{seq}},Z_{\text{func}},Z_{\text{length}}\right)=\prod_{t=1}^{L}p_{\theta }\left(x_{t}|x_{<t},Z_{\text{seq}},Z_{\text{func}},Z_{\text{length}}\right),$$

where $x_{<t}=\left(x_{1},\ldots ,x_{t-1}\right)$ and $Z_{\text{length}}$ represents a discrete length bin, enabling explicit control over sequence length.

#### Latent Priors

Each latent subspace in PLUM has a prior to encourage disentanglement and allow conditional control:

The sequence latent follows a standard normal,

(5) $$Z_{seq}∼𝒩(0,I),$$

capturing general sequence variability. The functional latent is conditioned on the functional label y and follows a Gaussian with parameters predicted by a learned MLP,

(6) $$Z_{\text{func}}∼𝒩(\mu_{\text{func}}(y),diag(\sigma_{\text{func}}^{2}(y))),$$

as in the CSVAE framework [15]. The length latent is conditioned on discrete length bin b∈{1,…,B},

(7) $$Z_{\text{length}}∼𝒩(\mu_{\text{length}}(b),diag(\sigma_{\text{length}}^{2}(b))),$$

allowing explicit control over peptide length while maintaining variability.

#### Latent Supervision and Adversarial Regularization

To enforce disentanglement, PLUM uses auxiliary prediction heads and a secondary decoder. The functional latent predicts antimicrobial activity via

(8) $$\overset{ˆ}{y}=g_{\text{func}}\left(Z_{\text{func}}\right),$$

and the length latent predicts the discrete length bin via

(9) $$\overset{ˆ}{b}=g_{\text{length}}\left(Z_{\text{length}}\right),$$

with b∈{1,…,B}.

A secondary decoder reconstructs sequences from the sequence latent alone,

(10) $$p_{\theta }\left(x|Z_{\text{seq}}\right),$$

encouraging $Z_{\text{seq}}$ to capture general sequence patterns independently of functional or length information. Adversarial predictors attempt to infer y and b from $Z_{\text{seq}}$, while the encoder is trained to minimize their accuracy. This forces $Z_{\text{seq}}$ to become invariant to functional and length information, ensuring it captures only general sequence patterns.

#### Training Objective

PLUM is trained with a multi-objective variational framework combining reconstruction, auxiliary supervision, latent regularization, and adversarial disentanglement.

##### Sequence reconstruction.

The primary decoder (Equation 4) reconstructs the full sequence from all latent subspaces:

(11) $${ℒ}_{\text{rec}}^{\text{full}}=-E_{q_{\phi }}\left[\log\;p_{\theta }\left(x|Z_{\text{seq}},Z_{\text{func}},Z_{\text{length}}\right)\right],$$

and a secondary decoder reconstructs using only the sequence latent (Equation 10):

(12) $${ℒ}_{rec}^{seq}=-E_{q_{\phi }}\left[\log\;p_{\theta }\left(x|Z_{seq}\right)\right].$$

##### Auxiliary supervision.

Functional and length latents are regularized via the auxiliary classifiers (Equation 8, Equation 9):

(13) $${ℒ}_{\text{func}}=CE\left(y,\overset{ˆ}{y}\left(Z_{\text{func}}\right)\right),\;{ℒ}_{\text{length}}={\left\|\ell -\hat{\ell }\left(Z_{\text{length}}\right)\right\|}_{2}^{2}.$$

##### Latent regularization.

All latent subspaces are regularized via KL divergence from their respective priors (Equation 5 to Equation 7):

(14) $${ℒ}_{KL}=\sum_{k\in \{\text{seq,func,length}\}}\;KL\left(q_{\phi }\left(Z_{k}|\cdot \right)\;\|\;p\left(Z_{k}|\cdot \right)\right).$$

##### Adversarial disentanglement.

Adversarial predictors on the sequence latent (Latent Supervision and Adversarial Regularization) attempt to infer y and b:

(15) $${ℒ}_{adv}=CE\left(y,{\overset{ˆ}{y}}_{adv}\left(Z_{seq}\right)\right)+{\left\|\ell -{\hat{\ell }}_{adv}\left(Z_{seq}\right)\right\|}_{2}^{2},$$

while the encoder is trained via gradient reversal to minimize their accuracy.

##### Total loss.

The full training objective is

(16) $${ℒ}_{\text{total}}={ℒ}_{\text{rec}}^{\text{full}}+\sum_{c\in \{\text{seq,}\;\text{func,}\;\text{length}\}}\lambda_{c}{ℒ}_{c}+\beta {ℒ}_{KL}-\gamma {ℒ}_{\text{adv}}$$

with weights $\lambda_{\text{seq}}$, $\lambda_{\text{func}}$, $\lambda_{\text{length}}$, β, γ controlling the contribution of each term.

### Training and Generation

PLUM is trained on the AMP Training data with the multi-objective variational framework (subsubsection 2.3.5). Sequences are one-hot encoded with START, STOP, and PAD tokens, and lengths are discretized into bins. Training jointly optimizes reconstruction, latent regularization, auxiliary functional and length supervision, and adversarial disentanglement of the sequence latent.

For generations, PLUM supports two modes: *de novo*, which samples latents from their priors conditioned on activity and length bins and generates sequences autoregressively; and prototype-conditioned, which encodes a peptide into the sequence latent, optionally perturbs it, and conditions functional and length latents to produce controlled analogues. Generated sequences are filtered for valid amino acids and target length. This enables exploration of peptide space, producing both novel sequences and targeted analogues with tunable functional and structural properties. Full training, hyperparameters, and generation details are in Supplementary Materials (Section S1.2, S1.3, S1.4), and ablation studies are reported in Section S3.

## Results

In this section, we comprehensively evaluate PLUM, focusing on both predictive performance and generative capabilities. We first assess the AMP identification and potency classifiers, establishing a basis for evaluating generated peptides. Next, we examine *de novo* peptide generation, analyzing functional fidelity, potency, and sequence diversity under controlled conditions. Finally, we test prototype-conditioned generation, evaluating PLUM’s ability to produce analogues with specified function and length while maintaining similarity to the original sequences. In all cases, PLUM is benchmarked against state-of-the-art generative models and baseline approaches to highlight its performance in controlled, interpretable peptide design.

### Evaluation of the classifiers

We initially focused on the design of AMP classifiers to evaluate our downstream model. These classifiers focused on general identification of AMPs vs. non-AMPs and evaluation of antimicrobial potency. By constructing these classifiers first, this enabled us to characterize PLUM-designed sequences later on. We evaluated our AMP classifier using two complementary approaches. First, the classifier was trained on the training set and evaluated on the held-out test set to assess generalization performance. Second, cross-validation on the training set was performed to estimate model stability and robustness. For benchmarking, test set results of our models were directly compared with established methods, including AMPLIFY, DIFF-AMP, and Peptide Scanner [33, 36, 17]. To enable fair comparison on the training data, we obtained classification results for all baseline models and used them alongside our cross-validation results as a consistent reference.

As summarized in Table 1, embedding-based models using ProtT5 and ESM2 consistently outperformed baselines across accuracy, precision, recall, and F1-score. ProtT5+MLP achieved the highest F1-score and precision, while ESM2+MLP achieved the highest accuracy and recall, demonstrating complementary strengths. These results indicate that pre-trained protein language model embeddings effectively capture sequence-level features critical for AMP identification. Based on overall performance, ProtT5+MLP was selected as the AMP Classifier.

For AMP potency prediction, ProtT5 embeddings were used exclusively to reduce model complexity while maintaining performance. As shown in Table 2, ProtT5-based classifiers performed comparably across models, with Random Forest achieving the best overall results (accuracy and F1-score 0.78). This model was chosen as the AMP Potency Classifier for subsequent analyses.

### Evaluation of De Novo Peptide Generation

PLUM enables *de novo* generation of peptide sequences by sampling from disentangled latent spaces, allowing control over functional attributes and sequence length. In this section, we evaluate conditioned generation, where peptides are explicitly generated for either antimicrobial or non-antimicrobial activity.

#### Experimental Setup

For conditioned generation, the sequence latent $Z_{\text{seq}}$ is sampled from a standard normal prior, while the functional $Z_{\text{func}}$ and length $Z_{\text{length}}$ latents are sampled from conditional priors corresponding to the desired activity and length. This enables controlled generation while maintaining sequence diversity.

Functional validity was assessed using the AMP Classifier with a probability threshold of 0.5. **AMP Yield** and **Non-AMP Yield** are defined as the fraction of generated sequences correctly classified according to the conditioning target. Predicted strength among AMP sequences is reported as **Potent AMP Yield**, capturing both antimicrobial identity and likely potency.

**Internal Diversity** is quantified as 1–⟨cosine similarity⟩ of peptide embeddings. Each peptide is embedded using ProtT5, pairwise cosine similarities are averaged, and subtracted from 1, with higher values indicating greater variation.

For benchmarking, PLUM was compared to AMP-GAN [32], DeanVAE [9], HydrAMP [30], MullerRNN [20], and two in-house cVAE baselines: Baseline 1 uses fully connected encoder/decoder networks, and Baseline 2 uses LSTM architectures to capture sequential dependencies. Prototype-conditioned generation for baselines was performed by perturbing the latent mean of a given peptide and decoding under the desired functional and length conditions. Detailed architectures, training procedures, and generation hyperparameters are provided in Supplementary Material (Section S2).

#### Results

Table 3 summarizes the performance of PLUM and baseline models for conditioned *de novo* peptide generation under both AMP and non-AMP settings. Reported metrics include the number of generated sequences, AMP/Non-AMP Yield, Potent AMP Yield, and internal diversity.

##### AMP Conditioned Generation

To generate AMPs, PLUM was conditioned with a functional latent value of 1, and peptide lengths were sampled uniformly between 5 and 35 amino acids. Using these settings, PLUM produced 45,000 sequences, achieving an AMP Yield of 0.885. Among these, 65.2% were predicted to have high antimicrobial potency (Potent AMP Yield), and internal diversity was 0.430, reflecting substantial sequence variation.

For comparison, we evaluated state-of-the-art generative models (AMP-GAN, DeanVAE, HydrAMP, MullerRNN) and two cVAE baselines implemented here (Baseline 1: fully connected cVAE; Baseline 2: LSTM cVAE), restricting all sequences to 5–35 amino acids. Baseline AMP Yields were generally lower than PLUM: AMP-GAN (0.456), DeanVAE (0.761), HydrAMP (0.827), Baseline 1 (0.658), and Baseline 2 (0.718). MullerRNN achieved the highest yield (0.937) but generated only 1,511 sequences, limiting large-scale comparability. Overall, PLUM produces large numbers of AMPs with high functional fidelity, substantial potency, and strong diversity, performing on par with or better than existing models.

##### Non-AMP Conditioned Generation

To generate non-AMPs, PLUM was conditioned with a functional latent value of 0, and peptide lengths were uniformly sampled between 5 and 35 amino acids. Using these settings, PLUM generated 45,000 sequences, achieving a Non-AMP Yield of 0.895 and internal diversity of 0.420, reflecting substantial variation among the sequences.

For comparison, HydrAMP achieved a Non-AMP Yield of 0.748, while the cVAE baselines reached 0.816 (Baseline 1) and 0.864 (Baseline 2). These results demonstrate that PLUM reliably produces large numbers of non-AMP sequences with strong functional fidelity and high diversity.

Further analysis of prototype-conditioned generation across different lengths shows that shorter sequences relative to the prototype tend to increase AMP yield, whereas longer sequences improve non-AMP yield (Supplementary Material, Section 3.3). Across all lengths, PLUM-generated sequences match the intended functional activity, maintain high antimicrobial potency when applicable, and retain diversity, performing as well as or better than existing generative models.

### Evaluation of Prototype-Conditioned Peptide Generation

PLUM is designed to generate peptide analogues of a given prototype, conditioned on both functional activity and length. To evaluate this capability, we used the AMP Test Data, which contains 521 sequences labeled as positive or negative AMPs.

#### Experimental Setup

For each prototype, sequences were generated by providing the prototype sequence as input to the model, specifying the functional attribute as either AMP or non-AMP, and explicitly defining the desired length.

To assess PLUM’s ability to generate AMP sequences, the functional condition was set to 1 (AMP) and lengths were sampled within ±7 residues of the prototype. For each prototype, 200 peptides were generated under these conditions. The same procedure was applied to two baseline models (Baseline1 and Baseline2), generating 200 peptides per prototype with identical functional and length constraints. Analogues were also generated using HydrAMP with the same prototypes, setting n_attempts to 200 and filtering_criteria to ‘discovery’. HydrAMP filters generated peptides based on classification results to retain only AMP sequences with high potency, as measured by MIC. Comparisons were further made with the Joker software [26], generating peptides for the same prototypes using its default parameters.

We also evaluated PLUM’s ability to generate non-AMP sequences, setting the functional condition to 0 (non-AMP) and sampling lengths within ±7 residues of the prototypes. For each prototype, 200 sequences were generated. Baseline1 and Baseline2 were evaluated using the same settings. Other baseline methods that do not support non-AMP generation were excluded from this evaluation.

All generated sequences were then evaluated using the AMP Classifier to determine which sequences are predicted as AMP or non-AMP for prototypes originally labeled as positive or negative. The AMP Potency Classifier was additionally used to quantify the number of potent AMPs among sequences predicted as AMPs. To assess how well generated sequences preserve prototype information, sequence similarity was measured between each generated peptide and its corresponding prototype using two established bioinformatic approaches: Needleman-Wunsch global alignment to assess overall similarity, and Smith-Waterman local alignment to assess conserved motifs, ensuring that the generated sequences are genuine analogues of the prototypes.

#### Results

We evaluated the ability of different methods to generate peptides matching the desired functional condition, using both AMP and non-AMP prototypes. Table 4 summarizes the total number of generated sequences that were predicted as AMPs or non-AMPs for each method, along with their potency and similarity to the original prototypes.

For generated AMPs, PLUM produced sequences from AMP and non-AMP prototypes, of which 30,606 and 7,164 were predicted as AMPs by the classifier, respectively. These numbers substantially exceed those for HydrAMP (23,837 and 3,789) and Joker (2,882 and 732). Baseline1 and Baseline2 generated far fewer sequences predicted as AMPs, reflecting their limited capacity for prototype-conditioned analogue generation. Among the predicted AMPs, PLUM also produced the largest number of potent sequences (21,659 and 6,327 for AMP and non-AMP prototypes, respectively), highlighting its ability to generate functionally active analogues even from non-AMP prototypes. Global and local similarity metrics confirm that the generated sequences maintain substantial resemblance to their prototypes, with PLUM achieving global similarity of 0.581 ± 0.065 for AMP prototypes and 0.536 ± 0.143 for non-AMP prototypes, and local similarity of 0.683 ± 0.059 and 0.621 ± 0.160, respectively.

For generated non-AMPs, PLUM produced 44,695 and 50,199 sequences from AMP and non-AMP prototypes, far exceeding Baseline1 (51, 11) and Baseline2 (66, 5). Global and local similarities indicate sequences remain close to prototypes, with PLUM achieving 0.584 ± 0.026 / 0.589 ± 0.023 (global) and 0.677 ± 0.017 / 0.681 ± 0.014 (local) for AMP and non-AMP prototypes, respectively. Overall, PLUM provides strong bidirectional functional control, generating both AMPs and non-AMPs while preserving prototype features. Shorter sequences relative to prototypes increase AMP yield, whereas longer sequences favor non-AMP yield (Supplementary Section 3.4).

## Discussion

Rising antimicrobial resistance (AMR) threatens both livestock and human health. In the U.S., prophylactic and growth-related antibiotic use in livestock was banned in 2017 to combat multidrug resistance [34], and AMR is projected to cause 1.91 million deaths per year by 2050 [7, 21]. AMPs offer protection against pathogens and boost immune responses [27, 24], making them a promising alternative. PLUM was developed to design and enhance AMPs for therapeutic use.

Upon its evaluation, we found that PLUM excelled at the generation of AMPs with superior diversity and breadth, and in tandem could easily generate non-AMP sequences at the same level. We generated 45,000 peptides from multiple models and found that PLUM achieved the highest AMP yield (0.885, Table 3), a 7% improvement over HydrAMP (0.827), while also increasing the average AMP diversity compared to HydrAMP (by 14%), surpassing other previously published models such as AMP-GAN and DeanVAE. In addition, PLUM generated non-AMP sequences with comparable complexity (Non-AMP Yield, 0.89), outperforming HydrAMP (Non-AMP Yield, 0.748) with a nearly 19% higher yield. For both outputs, PLUM demonstrated the ability to generate a large breadth of peptides across all lengths (5–35 AA, see Supplementary Section 3.3). We carried out additional analyses to assess the validity and reliability of PLUM-generated peptides, including their similarity to real peptide sequences and predicted toxicity. PLUM generations exhibited the lowest toxic tendencies and maintained high fidelity in amino acid composition (Supplementary Sections 3.5 and 3.6). This represents a major improvement over current models, and is a biologically important step forward for downstream experimental studies: the ability to design equally valid control sequences alongside a pool of diverse AMPs. As such, one of the major strengths of PLUM is its ability to effectively capture the underlying sequence representation of peptides that separates AMPs from Non-AMPs, a result that makes PLUM ideal for AMP generation.

Our results also demonstrate that PLUM can conditionally generate peptides based on existing AMP sequences or scaffolds, with the ability to adjust length and potency as required. PLUM could easily generate AMPs and non-AMP (control sequences) based on these metrics (Table 4, Supplementary Section 3.4) and outperformed models such as HydrAMP and Joker (37% more prototype-based AMPs than HydrAMP). Thus, an additional strength of PLUM is its ability to efficiently generate AMPs based on pre-existing scaffolds with greater diversity. Specific AMPs that have already been demonstrated to have effectiveness in various settings (cecropins, piscidins) can be further refined using PLUM. This will lead to the design of more stable, immune modulatory, and context-relevant AMPs. As such, PLUM represents a novel approach towards ML designed AMPs, with a distinct goal towards combating illness.

Another major advance in PLUM was the development of a broadly applicable AMP Potency Classifier. To evaluate PLUM’s AMP generation, we designed two classifiers: one for AMP identification (using ProT5 + MLP) and the other for overall antimicrobial potency (using ProT5 + RF, MIC estimation). While general AMP classifiers have been developed previously, classifiers that predict AMP MICs have so far been limited to development from datasets derived from *E. coli* [30]. While *E. coli* is of great importance to the microbial community, it by no means represents the vast diversity in physiology across bacteria. We find that our classifier can easily predict potent AMPs, and that PLUM designs a significantly higher number of these versus other models. We do note that incorporation of the wide variety of MIC data inherently decreases the AMP Potency Classifier’s metrics, but stress that this represents the biological diversity of incorporated values and reflects the nature of the data the classifier was trained on. As such, this represents the design of the first MIC classifier that can be applied toward any bacterial species. These classifiers enable the evaluation of strong predictive performance in downstream biological assays against a broad range of bacterial species and support the finding that PLUM can efficiently generate AMPs de-novo and based on existing sequences.

These results highlight PLUM’s superior ability to generate AMPs targeting diverse bacterial species. This a generalized tool that will allow for the generation of peptides for downstream analysis. The focus on designing shorter, linear AMPs means that peptides generated from the PLUM model will not suffer from scale-up issues, and will have increased stability and better oral bioavailability, as has been observed for other short AMPs [29]. We note that our model does not include any explicit biological filter, but focuses on large-scale generation of shorter peptides. Cost and stability are key for AMP adoption; PLUM enables the design of effective, affordable AMPs suitable for livestock and healthcare, reducing overall mortality.

## Supplementary Material

Supplement 1

## Figures and Tables

**Fig. 1.**
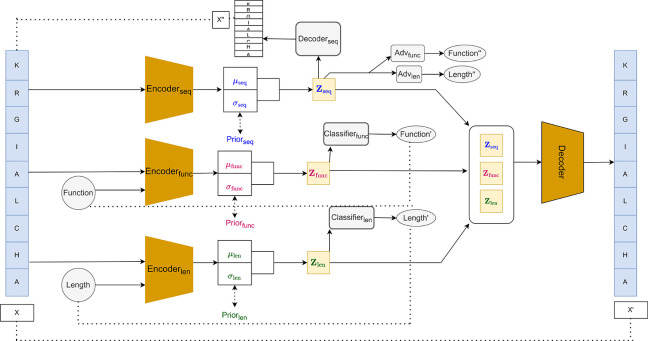
PLUM model architecture. Input peptide X is encoded into three disentangled latent spaces: $Z_{\text{seq}}$ (sequence patterns), $Z_{\text{func}}$ (functional activity), and $Z_{\text{length}}$ (peptide length), each with μ and σ outputs. KL divergence is computed with priors Prior_seq_, Prior_func_, Prior_length_ ($L_{\text{KL}}$). The main decoder reconstructs $X^{\prime }$ from all latents ($L_{\text{rec}}^{\text{full}}$), while a secondary decoder reconstructs $X^{\prime \prime }$ from $Z_{\text{seq}}$ alone ( $L_{\text{rec}}^{\text{seq}}$). Auxiliary classifiers predict function and length ($L_{\text{func}},L_{\text{length}}$), and adversarial heads predict function and length from $Z_{\text{seq}}\;\left(L_{\text{adv}}\right)$. Dotted lines indicate loss computations.

**Table 1.** AMP classification performance on training and test data.

Model	Train / CV (mean ± std)				Test			
	
	Accuracy	Precision	Recall	F1-score	Accuracy	Precision	Recall	F1-score

**ProtT5 + MLP**	**0.905 ± 0.004**	0.943 ± 0.005	**0.900 ± 0.006**	**0.906 ± 0.004**	**0.820**	0.840	**0.820**	**0.820**
ProtT5 + SVM	0.904 ± 0.004	0.943 ± 0.005	0.864 ± 0.006	0.902 ± 0.004	0.810	0.840	0.810	0.810
ProtT5 + RF	0.888 ± 0.003	0.932 ± 0.007	0.842 ± 0.008	0.885 ± 0.004	0.770	0.810	0.770	0.770
ESM2 + MLP	0.899 ± 0.004	0.911 ± 0.014	0.889 ± 0.014	0.900 ± 0.004	**0.820**	0.830	**0.820**	**0.820**
ESM2 + SVM	0.898 ± 0.004	0.942 ± 0.010	0.853 ± 0.006	0.895 ± 0.004	0.800	0.830	0.800	0.800
ESM2 + RF	0.887 ± 0.003	**0.945** ± 0.005	0.826 ± 0.008	0.881 ± 0.004	0.770	0.810	0.770	0.770
AMPLIFY	0.869	0.908	0.827	0.866	0.808	**0.879**	0.714	0.788
DIFF-AMP	0.808	0.861	0.743	0.798	0.719	0.833	0.547	0.660
Peptide Scanner	0.829	0.877	0.773	0.822	0.788	0.864	0.683	0.763

Metrics: accuracy, precision, recall, F1-score. For ProtT5 and ESM2 embeddings, training/CV results are mean ± std across folds; test results are on held-out set. Best values in each column are **bold**.

**Table 2.** Performance of ProtT5-based classifiers for AMP potency classification.

Model	Accuracy	Precision	Recall	F1-score

ProtT5 + SVM	0.76	0.76	0.76	0.75
**ProtT5 + RF**	**0.78**	**0.78**	**0.78**	**0.78**
ProtT5 + MLP	0.76	0.76	0.76	0.76

Metrics include accuracy, precision, recall, and F1-score. Best values in each column are highlighted in **bold**.

**Table 3.** Evaluation of *de novo* generated peptides.

Model	AMP				non-AMP		
	
	Generated	AMP Yield	Potent AMP Yield	Internal Diversity	Generated	non-AMP Yield	Internal Diversity

AMPGAN	45,000	0.456	0.651	0.337	-	-	-
DeanVAE	8,906	0.761	0.954	0.339	-	-	-
HydrAMP	45,000	0.827	0.264	0.377	45,000	0.748	0.425
MullerRNN	1,511	0.937	0.499	0.385	-	-	-
Baseline1	45,000	0.658	0.718	0.427	45,000	0.816	0.415
Baseline2	45,000	0.718	0.712	0.445	45,000	0.864	0.435
PLUM	45,000	0.885	0.652	0.430	45,000	0.895	0.420

AMP and non-AMP sequences were classified using an AMP Classifier (threshold 0.5). AMP Yield and non-AMP Yield denote the fraction of sequences matching the target function. Potent AMP Yield is the fraction of AMPs predicted to be highly potent. Internal diversity is 1 — 〈cosine similarity〉 of ProtT5 embeddings, with higher values indicating greater sequence variation.

**Table 4.** Generation performance for prototype-conditioned peptide generation.

Method	Prototype Function	Predicted AMPs/non-AMPs	Predicted Potent AMPs	Global Similarity	Local Similarity

**Generated AMPs**					

HydrAMP	AMP	23,837	7,655	0.640 ± 0.178	0.681 ± 0.170
HydrAMP	non-AMP	3,789	1,433	0.427 ± 0.266	0.478 ± 0.291
Joker	AMP	2,882	1,356	0.696 ± 0.245	0.696 ± 0.245
Joker	non-AMP	732	530	0.370 ± 0.395	0.370 ± 0.395
Baseline1	AMP	602	461	0.437 ± 0.185	0.594 ± 0.226
Baseline1	non-AMP	3	1	0.500 ± 0.000	0.732 ± 0.068
Baseline2	AMP	1,793	1,079	0.600 ± 0.132	0.737 ± 0.065
Baseline2	non-AMP	0	0	–	–
PLUM	AMP	30,606	21,659	0.581 ± 0.065	0.683 ± 0.059
PLUM	non-AMP	7,164	6,327	0.536 ± 0.143	0.621 ± 0.160

**Generated non-AMPs**					

Baseline1	AMP	51	–	0.286 ± 0.230	0.413 ± 0.318
Baseline1	non-AMP	11	–	0.366 ± 0.191	0.526 ± 0.268
Baseline2	AMP	66	–	0.268 ± 0.252	0.371 ± 0.346
Baseline2	non-AMP	5	–	0.427 ± 0.216	0.546 ± 0.279
PLUM	AMP	44,695	–	0.584 ± 0.026	0.677 ± 0.017
PLUM	non-AMP	50,199	–	0.589 ± 0.023	0.681 ± 0.014

Results are shown for both AMPs and non-AMPs, with metrics including total predicted AMPs/non-AMPs, predicted potent AMPs, and mean ± SD of global and local similarity to the prototypes.
