# Supplementary material for: A PLUM Job: Peptide modeLs for Understanding and engineering antiMicrobial therapeutics
Source: bioRxiv. 2026 Feb 23:2026.02.21.707214. Preprint. [Version 1] doi: 10.64898/2026.02.21.707214 (PMC13160118; doi:10.64898/2026.02.21.707214)
Supplement: Supplement 1 [file media-1.pdf]

Supplementary Material for "A PLUM Job: Peptide modeLs for Understanding and engineering antiMicrobial therapeutics"

Priyanka Banerjee, Iddo Friedberg, Britta Rued, Oliver Eulenstein

S1 PLUM data and model construction

S1.1 Extended Dataset Construction

To develop and evaluate the PLUM peptide generation model, we curated high-quality datasets of AMPs, non-AMPs, and associated potency data. These datasets enable both classification and generative modeling tasks, providing reliable ground truth for AMP identification, activity prediction, and prototype-conditioned peptide generation. Data were collected from multiple sources and carefully filtered for sequence length, linearity, and canonical amino acid composition, ensuring reproducibility and minimizing redundancy.

S1.1.1 Data sources

AMPs were compiled from four publicly available databases: CAMP, DBAASP, DRAMP, and GRAMPA [3, 4, 6, 10]. To construct a corresponding negative dataset of non-antimicrobial peptides, sequences with amino acid lengths between 5 and 35 residues were retrieved from UniProt. Sequences containing any of the following keywords were excluded: "Antimicrobial", "Antibiotic", "Antiviral", "Antifungal", "Fungicide", "Secreted", "Secretory", "Excreted", "Effector", "Defensin", "Disulfide bond", "Cross-link", "Antibacterial", "Bacteriostatic", and "Bactericidal", following the filtering strategy established in [9].

S1.1.2 AMP dataset

PLUM is designed to focus on linear AMPs, defined as peptides lacking cyclic or complex structures, disulfide bonds, or non-natural amino acid modifications, and restricted to a length of 5 to 35 amino acids (AA). This design choice reflects the goal of enabling rapid and scalable synthesis of novel PLUM-generated AMPs via solid phase peptide synthesis (SPPS), avoiding inaccessible AMP modifications from a synthesis standpoint [1, 5], and increasing proteolytic resistance and oral bioavailability via use of shorter sequences [8].

Consistent with these design constraints, the positive AMP dataset was compiled by combining sequences from four publicly available AMP databases: CAMP, DRAMP, DBAASP, and GRAMPA. Only natural peptides with lengths between 5 and 35 AA were retained. To exclude cyclic peptides, sequences explicitly labeled as cyclic in DBAASP and DRAMP were removed. Peptides from CAMP and GRAMPA were screened using AlphaFold2 structural predictions to identify and exclude sequences containing cyclic conformations or disulfide bridges. Disulfide bonds were identified by measuring the distance between sulfur atoms of cysteine residues; cysteine pairs with S–S distances below 2.5 Å were considered disulfide-linked. Cyclic peptides were detected by measuring the distance between the N- and C-terminal alpha carbons; sequences with head-to-tail distances below 2 Å were treated as cyclic [2, 11]. Following these filtering steps, the datasets contained 3,625,

2,888, 1,008, and 1,681 sequences from CAMP, DRAMP, DBAASP, and GRAMPA, respectively. The combined set of 9,202 sequences was subsequently screened against the UniProt database to identify peptides with annotated post-translational modifications (PTMs), and sequences with positive PTM records were removed. The resulting dataset was then further filtered to exclude peptides containing non-canonical amino acids and duplicate entries, yielding a final dataset of 4,723 unique linear AMP sequences.

S1.1.3 Non-AMP dataset

The negative dataset was filtered similarly, retaining sequences with lengths between 5 and 35 AAs and only including entries with protein- or transcript-level evidence in UniProt to ensure reliability. Linear peptides were identified using AlphaFold2, applying the same criteria for linearity as described for the AMP dataset, yielding 9,917 sequences. Sequences with annotated post-translational modifications (PTMs) were then removed based on UniProt records. Further filtering for valid amino acids and uniqueness produced 9,090 sequences. To create a balanced dataset, the negative set was adjusted to match both the size and length distribution of the positive set, resulting in a final set of 4,853 sequences (see Supplementary Material Figure S1).

S1.1.4 AMP MIC dataset

To enable classification of peptide activity, we compiled Minimum Inhibitory Concentration (MIC) data for AMPs from CAMP, DRAMP, DBAASP, and GRAMPA, considering sequences of 5–35 amino acids. The dataset was filtered to remove peptides containing non-canonical amino acids and duplicate sequences.

MIC is a standard measure of antimicrobial activity, representing the lowest concentration of a peptide that inhibits visible growth of a target microorganism. For each peptide, MIC values against all tested organisms were collected, standardized to μM, and averaged to provide a conservative estimate of broad-spectrum activity. Peptides with an average MIC ≤ 10 μM were labeled as active, while those with an average MIC > 10 μM were labeled as inactive. This threshold was selected based on classification performance during preliminary experiments, providing a balance between positive and negative classes and optimizing predictive accuracy for the AMP Potency Classifier.

These curated activity labels were subsequently used for training and evaluating the AMP Potency Classifier, which predicts whether a peptide is highly active or inactive.

S1.1.5 AMP Training and Test Dataset

We combined the curated AMP and non-AMP datasets to construct training and test sets for our classifier and generative models. Sequences were split using a length-aware, embedding-dissimilar strategy (see Supplementary Material Section 1.1.6), which preserves length distributions, minimizes similarity-driven data leakage, and balances positive and negative classes. The resulting training set contained 8,235 sequences (4,202 positive, 4,033 negative), and the test set contained 1,042 sequences (521 positive, 521 negative). The difference between total non-AMPs and the training and test non-AMPs reflects the exclusion of some non-AMP sequences to maintain length-aware, embedding-dissimilar splits. The training set was used to train both the generative model and the AMP classifier, whereas the test set was used to evaluate the AMP Classifier and to guide prototype-conditioned peptide generation.

S1.1.6 Training and Test Set Construction

To construct training and test sets for our classifier and generative models, we implemented a length-aware, embedding-dissimilar split to prevent similarity-driven data leakage.

Peptides were first grouped by sequence length to preserve realistic length distributions. Within each length group, a fraction of sequences was randomly selected as candidate test sequences. Each candidate's embedding (generated using ProtT5) was compared to all candidate training sequences in the same length bin using cosine similarity. Candidates were included in the final test set only if their similarity to every training sequence was below a threshold, set to the 98.4th percentile of pairwise similarities among positive (AMP) sequences. Candidates that did not meet this criterion remained in the training set.

After filtering, the test set was balanced across positive and negative classes, resulting in a training set of 8,235 sequences (4,202 positive, 4,033 negative) and a test set of 1,024 sequences (521 positive, 503 negative). This approach ensured that the test set contains length-matched peptides sufficiently distinct from training sequences, minimizing information leakage while maintaining representative length and class distributions.

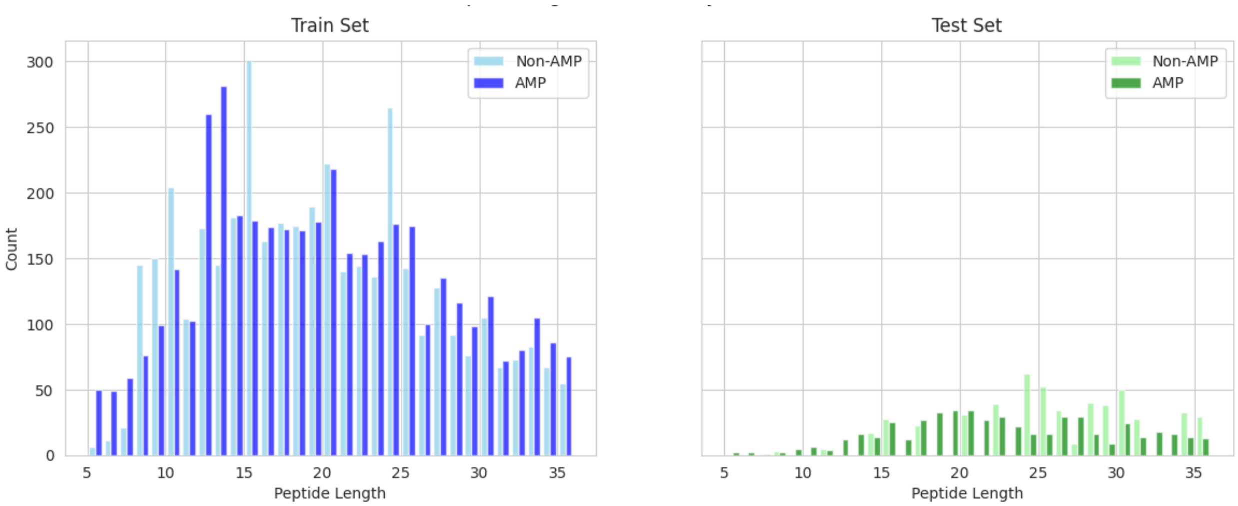

Figure S1: Distribution of AMPs and non-AMPs across different lengths in Train and Test datasets.

S1.2 Training Hyperparameters

PLUM is trained using a multi-objective conditional VAE framework with LSTM-based encoders and decoders. The training hyperparameters are summarized as follows:

- **Optimizer:** Adam
- **Learning rate:** 0.001
- **Batch size:** 64
- **Number of epochs:** 900
- **Hidden dimension:** 128
- **Latent dimensions:** $z = 8$, $w = 4$, $v = 4$

- **Conditioning dimension:** 1
- **Maximum sequence length:** 35

Loss weights. The multi-objective loss combines several terms:

- length_loss_weight = 1.0 (length reconstruction)
- func_loss_weight = 1.0 (functional classification)
- z_rec_weight = 1.0 (z-only reconstruction)
- kl_z_weight = 0.1 (KL divergence for sequence latent)
- adv_weight = 1.0 (adversarial disentanglement)

Training details. The decoder uses teacher forcing during training (`teacher_forcing=True`). KL divergence is applied to all latent variables (z, w, v) relative to their learned priors. The multi-objective loss also includes sequence reconstruction, z-only reconstruction, functional classification, length reconstruction, and adversarial disentanglement terms.

S1.3 Implementation Details

Software and Frameworks

PLUM was implemented in Python 3.10 using PyTorch 2.1. The LSTM encoder and decoder architectures were built with PyTorch's native modules. Auxiliary and adversarial heads were implemented as simple feedforward networks (MLPs) with ReLU activations. Training scripts, data preprocessing, and generation pipelines leverage NumPy, pandas, and standard scientific computing libraries.

Hardware

All experiments were performed on NVIDIA A100 GPUs with 40 GB memory. Model checkpointing and logging were handled using standard PyTorch utilities.

Data Preprocessing

Peptide sequences were one-hot encoded including `START`, `STOP`, and `PAD` tokens. Sequence lengths were discretized into B predefined bins. Functional labels (AMP vs non-AMP) were encoded as integer classes. Sequences exceeding the maximum length or containing non-standard amino acids were filtered out.

Model Architecture Details

The LSTM encoder and decoder each consist of 2 layers with 128 hidden units. Latent subspaces ($\mathbf{Z}_{\text{seq}}$, $\mathbf{Z}_{\text{func}}$, $\mathbf{Z}_{\text{length}}$) are 4-dimensional each. Auxiliary and adversarial heads are 2-layer MLPs with 128 hidden units.

Training Procedure

Models were trained using the Adam optimizer with a learning rate of 1×10^{-3} and a batch size of 64. Training ran for 900 epochs, depending on the experiment. The decoder uses teacher forcing (`teacher_forcing=True`). KL divergence is applied to all latent variables (z, w, v) relative to their learned priors. The multi-objective loss combines sequence reconstruction, z-only reconstruction, functional classification, length reconstruction, and adversarial disentanglement terms. Hyperparameters for the loss weights were selected empirically.

Generation Pipeline

For generation, latent variables are sampled from their respective priors. Prototype-conditioned generation perturbs the sequence latent around an existing peptide, while functional and length latents are set to desired values. The decoder generates sequences autoregressively, and sequences are filtered to ensure valid amino acid composition and adherence to target length bins.

S1.4 Generation Algorithms

PLUM supports two primary modes for generating peptide sequences: **De novo generation** and **Prototype-conditioned generation**. Both modes leverage the disentangled latent spaces to produce sequences with controllable functional and structural properties.

De Novo Generation

In de novo generation, sequences are produced without requiring a starting peptide. Latent variables are sampled from their respective priors: the sequence latent $\mathbf{Z}_{\text{seq}}$, the functional latent $\mathbf{Z}_{\text{func}}$, and the length latent $\mathbf{Z}_{\text{length}}$. Optional constraints on functional activity or length bins can be applied by conditioning the corresponding latent variables. Sequences are then generated autoregressively using the decoder. This mode enables exploration of novel peptide sequence space without relying on any existing peptide template.

Algorithm 1 De novo Peptide Generation

Require: Latent priors $p(\mathbf{Z}_{\text{seq}})$, $p(\mathbf{Z}_{\text{func}})$, $p(\mathbf{Z}_{\text{length}})$, optional functional label y and length bin b, decoder p_θ
Ensure: Generated peptide sequence $\mathbf{x}$

```
Sample sequence latent: Z_seq ~ p(Z_seq)
Sample functional latent: Z_func ~ p(Z_func | y)
Sample length latent: Z_length ~ p(Z_length | b)
Initialize empty sequence: x ← START token
while not STOP token or max length reached do
    Compute decoder logits: l_t = p_θ(x_<t, Z_seq, Z_func, Z_length)
    Optionally scale logits by temperature: l_t ← l_t/T
    Sample next amino acid: x_t ~ Softmax(l_t)
    Append x_t to sequence x
end while
return x
```

Prototype-Conditioned Generation

This algorithm generates peptide analogues conditioned on a *prototype sequence*. The prototype is first encoded into the sequence latent space $\mathbf{Z}_{\text{seq}}$, and optional Gaussian perturbations with magnitude σ allow exploration of nearby sequences. The functional latent $\mathbf{Z}_{\text{func}}$ is set according to the desired activity, while the length latent $\mathbf{Z}_{\text{length}}$ is sampled from its prior. Sequences are generated autoregressively by the decoder, with a soft bias β nudging the generated amino acids toward the prototype, balancing similarity and novelty. Hard length constraints (‘min_len‘ and ‘max_len‘) ensure valid sequence lengths, and a temperature parameter can control stochasticity during sampling. This approach enables controlled generation of sequences similar to a prototype while allowing local diversity, with key hyperparameters including σ (perturbation magnitude), β (prototype bias), minimum and maximum lengths, temperature, and stochasticity of sampling.

Algorithm 2 Prototype-Conditioned Peptide Generation

Require: Prototype peptide $\mathbf{x}_{\text{proto}}$, latent encoder q_ϕ, functional label y, perturbation magnitude σ, soft bias β, min/max lengths, decoder p_θ
Ensure: Generated peptide sequence $\mathbf{x}$
Encode prototype: $\mathbf{Z}_{\text{seq}}^{\text{proto}} \sim q_\phi(\mathbf{Z}_{\text{seq}} \mid \mathbf{x}_{\text{proto}})$
Optionally perturb: $\mathbf{Z}_{\text{seq}} \leftarrow \mathbf{Z}_{\text{seq}}^{\text{proto}} + \epsilon$, $\epsilon \sim \mathcal{N}(0, \sigma^2)$
Set functional latent: $\mathbf{Z}_{\text{func}} \sim p(\mathbf{Z}_{\text{func}} \mid y)$
Set length latent: $\mathbf{Z}_{\text{length}} \sim p(\mathbf{Z}_{\text{length}})$
Initialize sequence with START token: $\mathbf{x} \leftarrow$ START
while not STOP token or max length reached **do**
    Compute decoder probabilities: $\mathbf{p}_t = p_\theta(x_t \mid \mathbf{x}_{<t}, \mathbf{Z}_{\text{seq}}, \mathbf{Z}_{\text{func}}, \mathbf{Z}_{\text{length}})$
    Apply soft prototype bias: $\mathbf{p}_t \leftarrow \beta \cdot \mathbf{x}_t^{\text{proto}} + (1-\beta) \cdot \mathbf{p}_t$
    Sample or select next amino acid x_t from $\mathbf{p}_t$
    Append x_t to sequence $\mathbf{x}$
end while
return x

Key hyperparameters used in our experiments included a perturbation magnitude $\sigma = 0.01$, prototype bias $\beta = 0.30$, minimum and maximum lengths $[5, 35]$, and a sampling temperature $T = 1.0$. Stochastic sampling was enabled to allow diverse sequence generation.

S2 Baseline Methods

S2.1 Baseline 1: Conditional Sequence Variational Autoencoder (cVAE)

This baseline employs a conditional variational autoencoder (cVAE) to generate peptide sequences conditioned on functional activity and sequence length. Peptides are represented as one-hot encoded sequences, with variable lengths handled via masking. Conditioning variables are explicitly incorporated into both the encoder and decoder.

S2.1.1 Model Architecture

The model consists of a feedforward encoder–decoder architecture. The encoder maps an input peptide sequence $\mathbf{x}$ and a conditioning vector $\mathbf{c}$ to a latent Gaussian distribution parameterized by a mean $\boldsymbol{\mu}$ and log-variance $\log \boldsymbol{\sigma}^2$. The conditioning vector comprises two components: (i) a binary functional label and (ii) a normalized peptide length.

The encoder flattens the one-hot encoded peptide sequence and concatenates it with the conditioning vector before passing it through fully connected layers with ReLU activations. The decoder mirrors this structure, taking a sampled latent vector $\mathbf{z}$ concatenated with the same conditioning vector and mapping it to a sequence of amino acid logits. The output is reshaped to produce position-wise categorical distributions over the amino acid vocabulary, with padding positions masked during training.

S2.1.2 Latent Variable Formulation

Latent variables are sampled using the reparameterization trick:

$$\mathbf{z} = \boldsymbol{\mu} + \boldsymbol{\epsilon} \odot \boldsymbol{\sigma}, \quad \boldsymbol{\epsilon} \sim \mathcal{N}(\mathbf{0}, \mathbf{I}).$$

For prototype-conditioned generation, the latent mean $\boldsymbol{\mu}$ of a peptide is used deterministically, and small Gaussian perturbations are applied to explore structurally related variants.

S2.1.3 Training Objective

The model is trained by minimizing a weighted sum of a reconstruction loss and a Kullback–Leibler (KL) divergence term:

$$\mathcal{L} = \mathcal{L}_{\text{rec}} + \lambda \, \mathcal{L}_{\text{KL}},$$

where $\lambda = 0.01$ in our experiments. The reconstruction loss is a categorical cross-entropy over amino acids at each sequence position, ignoring padded positions. The KL divergence regularizes the latent space toward a standard normal prior.

S2.1.4 De Novo Peptide Generation

Peptides are generated *de novo* by sampling latent vectors from the prior distribution and decoding them under specified functional and length conditions. The decoded sequences are truncated to the desired length, and amino acids are sampled independently at each position from the softmax-normalized outputs. Temperature scaling can be applied to modulate sequence diversity.

Algorithm 3 De Novo Peptide Generation with cVAE

Require: Trained decoder D, latent dimension L, target length s, functional label f, temperature T

1: Sample latent vector $z \sim \mathcal{N}(0, I^L)$
2: Create conditioning vector $c = [f, s/\text{max\_seq\_len}]$
3: Decode logits: $x_{\text{logits}} = D(z, c)$
4: Truncate logits to target length: $x_{\text{logits}} = x_{\text{logits}}[: s, :]$
5: Apply softmax with temperature T and sample amino acids: $x \sim \text{Softmax}(x_{\text{logits}}/T)$
6: **return** generated peptide sequence x

S2.1.5 Prototype-Conditioned Peptide Generation

To generate peptide analogues, each prototype is first encoded to obtain its latent mean. Small Gaussian noise is added to this latent vector, which is then decoded under different combinations of functional labels and target lengths. By systematically varying functional and length conditions, this approach produces diverse analogues while preserving similarity to the original sequence. Multiple analogues per condition are generated to increase coverage of the conditioned sequence space.

Algorithm 4 Prototype-Conditioned Peptide Generation with cVAE

Require: Trained encoder E, decoder D, prototype peptide sequence x_{proto}, prototype functional label f_{proto}, prototype length s_{proto}, target functional label f_{target}, target length s_{target}, latent dimension L, temperature T, perturbation standard deviation σ

1: Encode prototype: $\mu, \log \sigma^2 = E(x_{\text{proto}}, [f_{\text{proto}}, s_{\text{proto}}/\text{max\_seq\_len}])$
2: Compute latent vector with perturbation: $z = \mu + \epsilon$, where $\epsilon \sim \mathcal{N}(0, \sigma^2 I^L)$
3: Create target conditioning vector $c = [f_{\text{target}}, s_{\text{target}}/\text{max\_seq\_len}]$
4: Decode logits: $x_{\text{logits}} = D(z, c)$
5: Truncate logits to target length: $x_{\text{logits}} = x_{\text{logits}}[: s_{\text{target}}, :]$
6: Apply softmax with temperature T and sample amino acids: $x \sim \text{Softmax}(x_{\text{logits}}/T)$
7: **return** generated peptide analogue x

Model, Training, and Generation Hyperparameters The cVAE model was trained with a latent dimension of $L = 8$ and hidden layers of 128 units in both the encoder and decoder. Peptides were represented as one-hot encoded sequences of 20 amino acids, with variable-length sequences padded to the maximum length in the dataset. The model was optimized using the Adam optimizer with a learning rate of 1×10^{-3} and a batch size of 16 over 500 epochs. The training objective consisted of a masked categorical cross-entropy reconstruction loss and a KL divergence term weighted by $\lambda = 0.01$.

During peptide generation, conditioning vectors combined the functional label and normalized sequence length. De novo peptides were generated by sampling latent vectors from the prior with temperature $T = 1.0$, whereas prototype-conditioned analogues were generated by encoding the prototype, adding Gaussian perturbations to the latent vector with standard deviation $\sigma = 0.01$, and decoding under the target function and length with temperature $T = 1.0$. Amino acids were sampled independently at each sequence position for both generation modes.

S2.2 Baseline 2: Conditional Sequence Variational Autoencoder with LSTM (cVAE LSTM)

This baseline employs a conditional variational autoencoder (cVAE) with recurrent neural networks to generate peptide sequences. Peptides are represented as one-hot encoded sequences augmented with special tokens for start-of-sequence (SOS), end-of-sequence (EOS), and padding (PAD). Variable-length sequences are handled via padding and packed LSTM sequences. Conditioning variables, comprising the functional label and normalized peptide length, are incorporated into both the encoder and decoder.

S2.2.1 Model Architecture

The encoder is a single-layer LSTM that processes packed peptide sequences. The final hidden state is concatenated with the conditioning vector and passed through linear layers to produce the latent mean $\boldsymbol{\mu}$ and log-variance $\boldsymbol{\log \sigma^2}$. The decoder is an autoregressive LSTM with an embedding layer, which generates one token at a time. Its initial hidden state is computed from the concatenation of the latent vector and conditioning vector. At each time step, the decoder outputs logits over the amino acid vocabulary plus special tokens, and the next token is sampled either from the target sequence (teacher forcing) or from the decoder output.

S2.2.2 Latent Variable Formulation

Latent variables are sampled using the reparameterization trick:

$$\mathbf{z} = \boldsymbol{\mu} + \boldsymbol{\epsilon} \odot \boldsymbol{\sigma}, \quad \boldsymbol{\epsilon} \sim \mathcal{N}(\mathbf{0}, \mathbf{I}).$$

During prototype-conditioned generation, the latent mean $\boldsymbol{\mu}$ is used deterministically, with small Gaussian perturbations applied to generate structurally related analogues.

S2.2.3 Training Objective

The model is trained by minimizing a weighted sum of a masked categorical cross-entropy reconstruction loss and a KL divergence regularization term:

$$\mathcal{L} = \mathcal{L}_{\text{rec}} + \lambda \, \mathcal{L}_{\text{KL}},$$

with $\lambda = 0.01$. The reconstruction loss ignores padded positions. Teacher forcing is applied during training, with a ratio decaying from 1.0 to 0.1 over epochs. The model is optimized using the Adam optimizer with a learning rate of 1×10^{-3}, a batch size of 16, and trained for 500 epochs.

S2.2.4 De Novo Peptide Generation

Peptides are generated *de novo* by sampling latent vectors from the prior distribution and decoding them autoregressively under specified functional and length conditions. Generation stops when either the EOS token is sampled or the target length is reached. Temperature scaling controls the diversity of sampled sequences.

Algorithm 5 De Novo Peptide Generation with cVAE LSTM

Require: Trained decoder D, latent vector z, target length s, functional label f, temperature T

Create conditioning vector $c = [f, s/D.max\_len]$
Decode sequence autoregressively with decoder $D(z, c)$
Truncate to target length or stop at EOS token
Apply softmax with temperature T and sample amino acids
return generated peptide sequence

S2.2.5 Prototype-Conditioned Peptide Generation

To generate peptide analogues, each prototype sequence is encoded with the LSTM encoder to obtain its latent mean $\boldsymbol{\mu}$. Small Gaussian noise is added to this latent vector, which is then decoded autoregressively under target functional and length conditions. This procedure produces diverse analogues while preserving similarity to the prototype.

Algorithm 6 Prototype-Conditioned Peptide Generation with cVAE LSTM

Require: Trained encoder E, decoder D, prototype peptide x_{proto}, prototype function f_{proto}, prototype length s_{proto}, target function f_{target}, target length s_{target}, latent dimension L, temperature T, perturbation std σ

1: Encode prototype: $\mu, \log \sigma^2 = E(x_{\text{proto}}, [f_{\text{proto}}, s_{\text{proto}}/D.max\_len])$
2: Perturb latent vector: $z = \mu + \epsilon$, $\epsilon \sim \mathcal{N}(0, \sigma^2 I^L)$
3: Create target conditioning vector $c = [f_{\text{target}}, s_{\text{target}}/D.max\_len]$
4: Decode sequence autoregressively with decoder $D(z, c)$
5: Truncate to target length or stop at EOS token
6: Apply softmax with temperature T and sample amino acids
7: **return** generated peptide analogue

Model, Training, and Generation Hyperparameters The cVAE LSTM was trained with a latent dimension of $L = 16$, LSTM hidden size of 128, and embedding dimension of 64 in the decoder. Peptides were represented with one-hot encoding over 20 amino acids plus SOS, EOS, and PAD tokens, padded to the maximum sequence length. The optimizer was Adam with a learning rate of 1×10^{-3}, batch size 16, over 500 epochs. The KL divergence weight was $\lambda = 0.01$, and teacher forcing decayed from 1.0 to 0.1 during training.

During generation, conditioning vectors combined the functional label and normalized sequence length. De novo peptides were generated with temperature $T = 1.0$, while prototype-conditioned analogues used Gaussian perturbations with $\sigma = 0.01$ and temperature $T = 1.0$. Amino acids were sampled independently at each step, stopping at EOS or the target length.

Table S1: Comparison of Baseline 1 and Baseline 2 cVAE models

**Feature**	**Baseline 1**	**Baseline 2**
Encoder	Fully connected feedforward	Single-layer LSTM (packed sequences)
Decoder	Fully connected feedforward	Autoregressive LSTM with embedding
Sequence representation	One-hot (20 AAs)	One-hot + SOS/EOS/PAD (23 tokens)
Conditioning	Functional label + normalized length	Functional label + normalized length
Latent dimension	8	16
KL weight (λ)	0.01	0.01
Teacher forcing	N/A	Decaying from 1.0 $\rightarrow$ 0.1
Generation mode	Parallel, per-position	Stepwise autoregressive, stop at EOS or target length
Prototype-conditioned generation	Add Gaussian noise ($\sigma = 0.01$)	Add Gaussian noise ($\sigma = 0.01$)
Training epochs	500	500
Batch size	16	16
Optimizer	Adam (LR 1×10^{-3})	Adam (LR 1×10^{-3})

S3 Supplementary Analysis

S3.1 Ablation of Training Loss Components

To evaluate the contribution of individual training losses to PLUM's performance, we conducted an ablation study in which specific components of the full loss function were removed. The full training loss is defined as:

$$\mathcal{L}_{\text{total}} = \mathcal{L}_{\text{rec}}^{\text{full}} + \lambda_{\text{seq}}\mathcal{L}_{\text{rec}}^{\text{seq}} + \lambda_{\text{func}}\mathcal{L}_{\text{func}} + \lambda_{\text{length}}\mathcal{L}_{\text{length}} + \beta\mathcal{L}_{\text{KL}} - \gamma\mathcal{L}_{\text{adv}}. \quad (1)$$

We systematically removed individual terms to assess their impact on peptide generation quality. Generated sequences were evaluated using a separate AMP classification model, reporting Accuracy, F1 score, Precision, and Recall. Additionally, reconstruction loss ($\mathcal{L}_{\text{rec}}^{\text{full}}$) was recorded as a measure of sequence fidelity. The results of this ablation study are summarized in Table S2, showing the impact of removing individual loss components on sequence reconstruction and AMP classification performance.

Table S2: Ablation study of PLUM training losses. Metrics were computed using an external AMP classifier on 10,000 generated sequences per functional condition.

Loss Configuration	Reconstruction loss	Accuracy	F1	Precision	Recall
Full model	1.5594	0.9086	0.9076	0.9178	0.8976
No z-only decoder	1.8263	0.8569	0.8557	0.8634	0.8480
No adversarial	1.6541	0.9071	0.9042	0.9335	0.8767
No z-only decoder + No adversarial	1.6826	0.8691	0.8623	0.9099	0.8194
No functional loss	1.5567	0.8547	0.8509	0.8739	0.8291
No length loss	1.6180	0.8256	0.8092	0.8928	0.7400
No functional & length loss	1.5543	0.8467	0.8403	0.8768	0.8067

S3.2 Ablation of Latent Subspaces

To assess the contribution of each disentangled latent subspace in PLUM, we performed an ablation study comparing models trained with selective combinations of latent variables:

- **Z + W only:** Retains sequence ($\mathbf{Z}_{\text{seq}}$) and functional ($\mathbf{Z}_{\text{func}}$) latents; length latent excluded.
- **Z + V only:** Retains sequence and length ($\mathbf{Z}_{\text{length}}$) latents; functional latent excluded.
- **Full Z + W + V:** Baseline model with all three latent subspaces.

Generated sequences from each configuration were evaluated using reconstruction loss ($\mathcal{L}_{\text{rec}}^{\text{full}}$) and AMP classification metrics (Accuracy, F1, Precision, Recall), consistent with the evaluation in the training loss ablation study. The results are summarized in Table S3.

Excluding the functional latent ($\mathbf{Z}_{\text{func}}$) reduces functional specificity, while removing the length latent ($\mathbf{Z}_{\text{length}}$) leads to poorer length control. The sequence latent ($\mathbf{Z}_{\text{seq}}$) is essential for high-fidelity sequence reconstruction. Overall, the full Z + W + V configuration achieves the best balance of reconstruction accuracy and functional predictability, confirming that all three latent subspaces provide complementary information critical for interpretable and controllable peptide generation.

Table S3: Ablation of latent subspaces. Excluding specific latents affects functional or length control.

Latent Configuration	Reconstruction loss	Accuracy	F1	Precision	Recall
Z + W only	1.5234	0.8471	0.8415	0.8732	0.8120
Z + V only	1.5564	0.8689	0.8665	0.8825	0.8510
Full Z + W + V	1.5594	0.9086	0.9076	0.9178	0.8976

S3.3 Length-wise Analysis of De Novo Peptide Generation

PLUM shows the most consistent and robust AMP generation across peptide lengths, with yields above 0.75 for short sequences (5–10 residues) and moderate to high values (> 0.6) for longer peptides, while producing substantial counts at nearly all lengths. Other models are less consistent: AMPGAN exhibits a sharp decline at medium and long lengths; Baseline1 and Baseline2 fluctuate despite high sequence counts; MullerRNN maintains high yield but generates very few peptides per length; DeanVAE sustains yield unevenly, dominated by certain lengths; and HydrAMP performs well for short sequences but declines with elongation. Overall, PLUM combines high yield with broad length coverage, outperforming other models in both consistency and sequence count (See Figure S2).

For non-AMP generation across peptide lengths, PLUM shows consistently high yield, typically above 0.7 from length 8 residues onward, reaching $\sim$0.95 for medium-length peptides (12–25 residues), with substantial sequence counts across nearly all lengths. HydrAMP shows increasing non-AMP yield with length, starting low for very short sequences ($\sim$0.1–0.28) and reaching $\sim$0.75–0.8 for longer peptides, though very short lengths are underrepresented. Baseline1 and Baseline2 display more variable yields, starting low at short lengths and rising to > 0.85 for longer sequences, reflecting less control over early-length non-AMP generation. (See Figure S3).

Overall, PLUM demonstrates the most consistent and robust non-AMP generation across lengths, complementing its strong AMP performance, while other models show either fluctuations or sparse coverage at certain lengths.

S3.4 Length-wise Analysis of Prototype-Conditioned Generation

To further evaluate the prototype-conditioned peptide generation, we assessed how the lengths of generated sequences compared to their prototypes. For each prototype, generated analogues were analyzed in terms of *directional length difference*:

$$\text{Length difference} = \text{generated sequence length} - \text{prototype length}$$

Length differences were binned into signed intervals (step size of 2 residues) to capture whether sequences were shorter or longer than the prototype. For each bin, we summarized:

- The total number of generated sequences.
- The number of sequences predicted to be antimicrobial (AMP) and non-antimicrobial (non-AMP) using a classifier with a threshold of 0.5.
- The fraction of sequences predicted as AMP or non-AMP within each bin (yield).

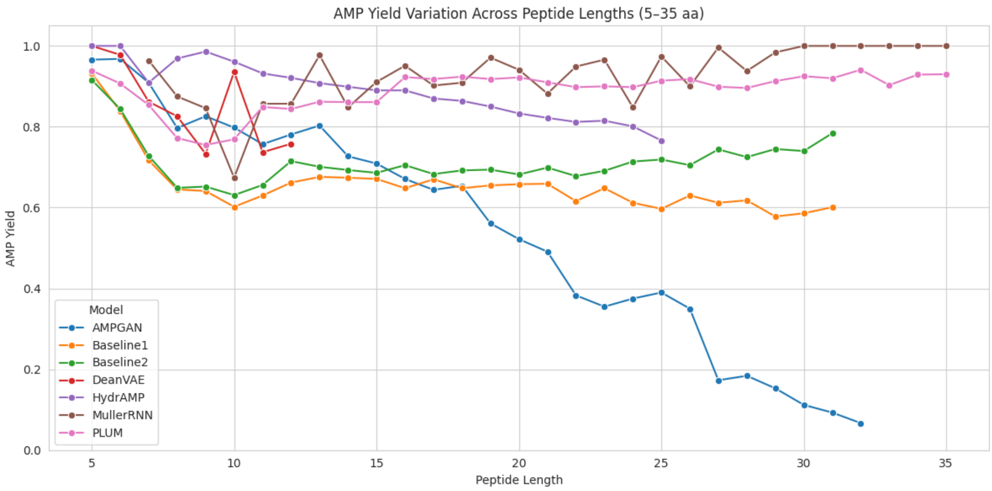

Figure S2: AMP yield across peptide lengths (5–35 residues) for different generative models. PLUM maintains consistently high AMP yield across nearly all lengths, while other models show more variability.

This analysis provides a detailed view of how prototype-conditioned generation affects sequence length distribution and predicted activity, highlighting whether certain length deviations are more likely to produce AMP or non-AMP sequences.

For AMP prototypes, the predicted AMP fraction is highest for sequences substantially shorter than the prototype (e.g., <-10 bin, 0.814) and decreases as sequences approach or slightly exceed the prototype length (0 to +2 bin, 0.615). Notably, the AMP fraction shows a modest recovery in the +2 to +4 bin (0.655) before gradually declining in longer bins, reaching 0.500 in the >+10 bin. In contrast, non-AMP yield exhibits the opposite trend, increasing with sequence length from 0.332 (<-10) to 0.920 (>+10). These patterns suggest that shorter analogues preferentially retain AMP activity, whereas elongation increases the likelihood of non-AMP prediction (See Figure S4). For non-AMP prototypes, AMP yields remain low across all length bins, with minor peaks in the <-10 (0.596) and >+10 (0.361) bins, while non-AMP fractions are consistently high (> 0.58 across all bins). This indicates that prototype-conditioned generation preserves the non-antimicrobial character of these sequences regardless of length deviations. (See Figure S5).
Overall, these results demonstrate that sequence length relative to the prototype modulates predicted activity: shorter sequences favor AMP activity for AMP prototypes, whereas longer sequences increase non-AMP likelihood, while non-AMP prototypes remain largely resistant to AMP conversion across all length variations.

S3.5 Amino Acid Composition Analysis of Generated Peptides

To evaluate the biochemical fidelity of our generative model **PLUM**, we compared the amino acid composition of its generated peptides to the combined Train+Test AMP dataset, alongside other generative models including AMPGAN, HydrAMP, MullerRNN, DeanVAE, Baseline1, and Baseline2. The total absolute deviation from the reference Train+Test AMP composition was calculated across all 20 amino acids, with PLUM showing a moderate deviation of approximately 19.7%, compared to HydrAMP (77.2%) and MullerRNN (25.0%), which exhibited the largest deviations, while Baseline1 (5.8%) and Baseline2 (7.4%) were closest to the reference (See Figure S7. PLUM reproduces the frequencies of common residues in natural AMPs, including Lysine (K, 12.3%), Leucine

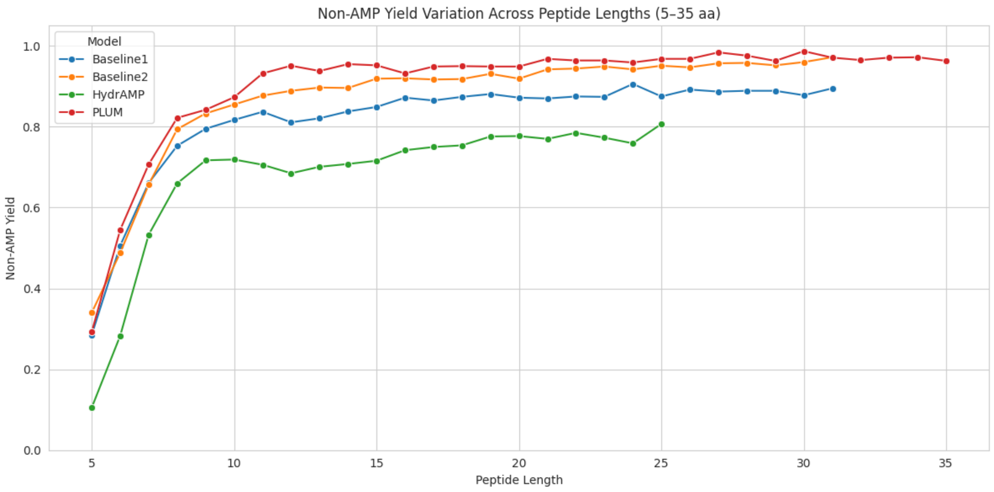

Figure S3: Non-AMP yield across peptide lengths (5–35 residues) for different generative models. PLUM exhibits consistently high non-AMP yield across lengths.

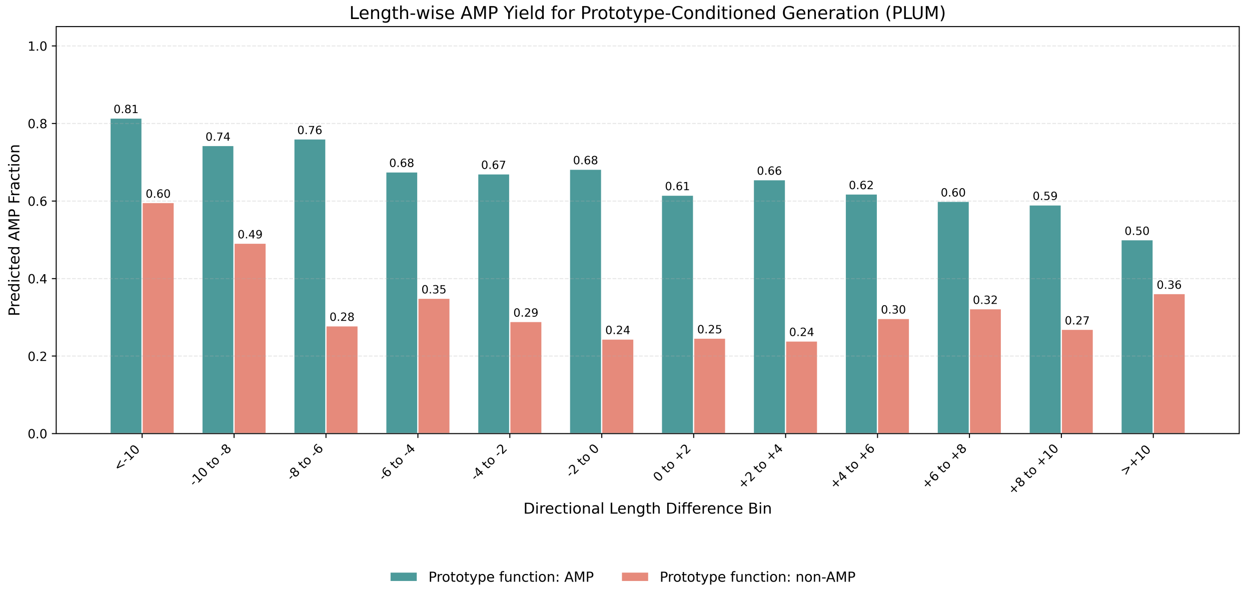

Figure S4: Length-wise AMP yield for AMP prototype and non-AMP prototype in prototype-conditioned peptide generation. Bar plots show the predicted fraction of AMP sequences across signed length difference bins.

(L, 13.2%), Valine (V, 6.1%), and Glycine (G, 15.0%), with slight overrepresentation of Glycine and Alanine, while rare residues such as Tryptophan (W, 1.44%) and Tyrosine (Y, 1.19%) remain low. In contrast, HydrAMP strongly overrepresents W (25.5%) and R (16.6%), whereas AMPGAN and DeanVAE show larger deviations in less frequent residues (See Figure S6. Overall, PLUM demonstrates a favorable balance between fidelity and diversity, producing sequences that closely resemble natural AMPs while allowing for variability, suggesting suitability for downstream functional and toxicity analyses.

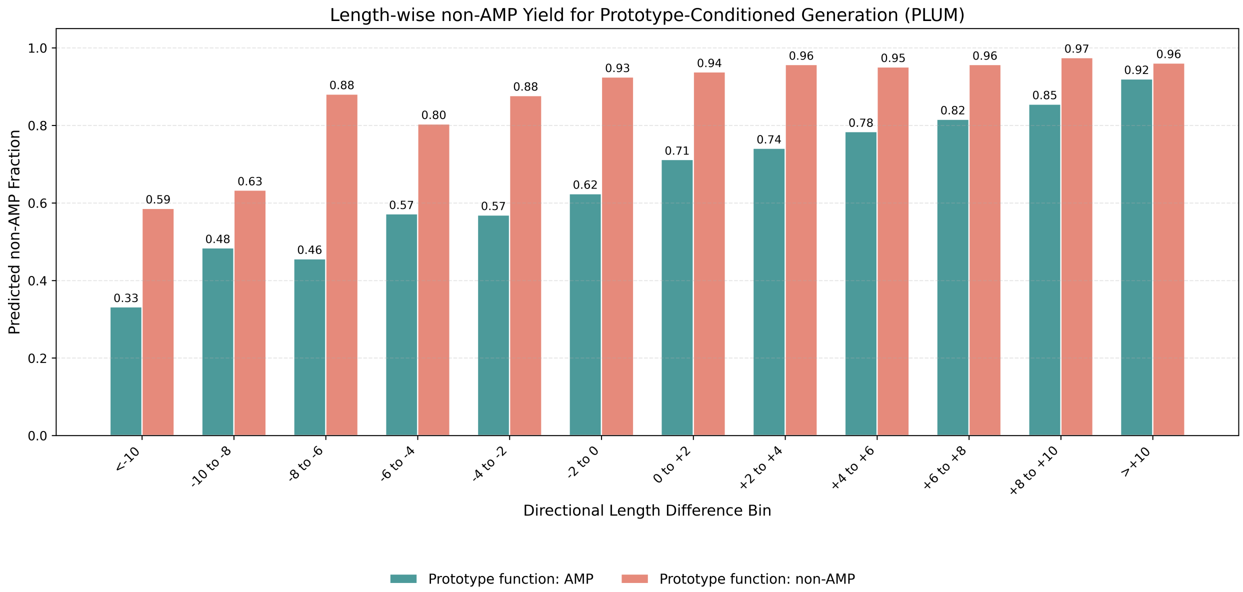

Figure S5: Length-wise non-AMP yield for AMP prototype and non-AMP prototype in prototype-conditioned peptide generation. Bar plots show the predicted fraction of non-AMP sequences across signed length difference bins.

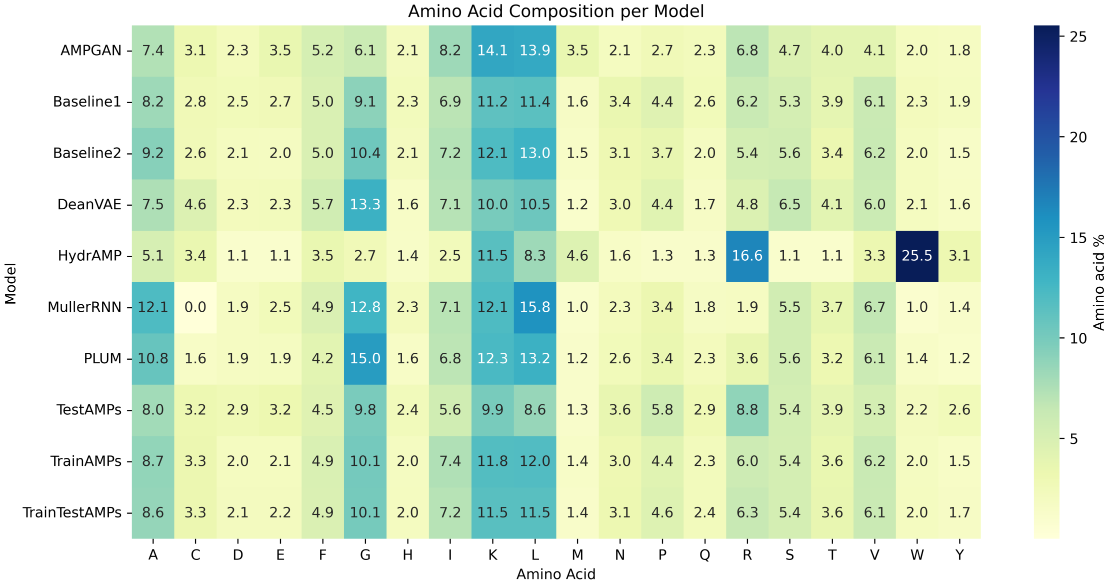

Figure S6: Amino acid composition of peptides generated by different models and the combined Train+Test AMP dataset. PLUM reproduces the overall residue distribution closely while maintaining diversity.

S3.6 Predicted Toxicity of Generated Peptides

To evaluate the safety of the generations from our model, all *de novo* generated AMPs from the generative models were analyzed using ToxinPred3 [7]. The fraction of predicted toxic sequences varied among models. Notably, **PLUM** generated only approximately 21% toxic peptides, the

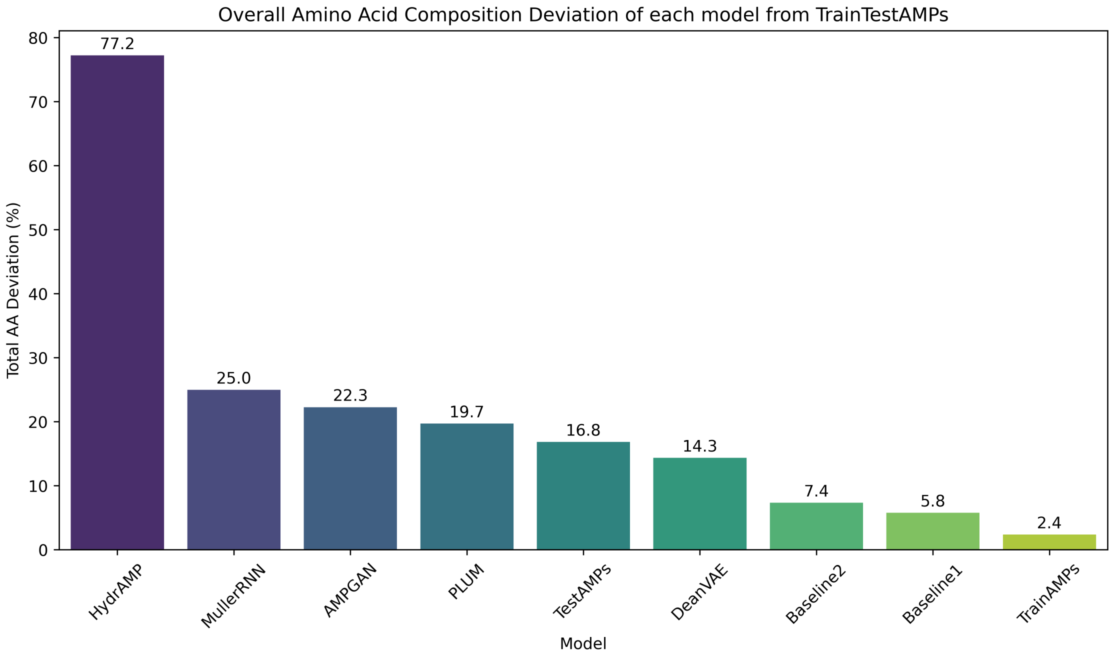

Figure S7: Total amino acid composition deviation of each model from the combined Train+Test AMPs. PLUM shows moderate deviation compared to other generative models, indicating a good balance between fidelity and variability.

lowest among all models. In contrast, HydrAMP generated nearly 50% toxic peptides, DeanVAE approximately 40%, and other models including AMPGAN, Baseline1, and Baseline2 around 25%. These results demonstrate that PLUM is capable of generating novel peptides that are both diverse and relatively safe, supporting its suitability for downstream functional and therapeutic applications (See Figure S8).

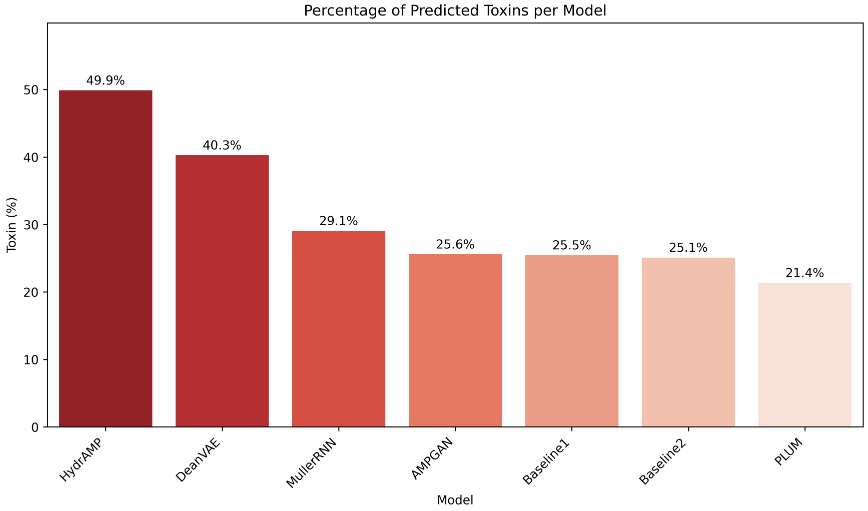

Figure S8: Percentage of predicted toxic peptides for *de novo* generated sequences from each model. PLUM exhibits the lowest predicted toxicity among all generative models.

References

[1] Ahmer Bin Hafeez, Xukai Jiang, Phillip J. Bergen, and Yan Zhu. Antimicrobial peptides: An update on classifications and databases. *International Journal of Molecular Sciences*, 22(21):11691, 2021.

[2] Ivana Bošnjak, Viktor Bojović, Tanja Šegvić-Bubić, and Ana Bielen. Occurrence of protein disulfide bonds in different domains of life: a comparison of proteins from the protein data bank. *Protein engineering, design & selection*, 27(3):65–72, 2014.

[3] Ulka Gawde, Shuvechha Chakraborty, Faiza Hanif Waghu, Ram Shankar Barai, Ashlesha Khanderkar, Rishikesh Indraguru, Tanmay Shirsat, and Susan Idicula-Thomas. Campr4: a database of natural and synthetic antimicrobial peptides. *Nucleic Acids Research*, 51(D1):D377–D383, 2023.

[4] Tianyue Ma, Yanchao Liu, Bingxin Yu, Xin Sun, Huiyuan Yao, Chen Hao, Jianhui Li, Maryam Nawaz, Xun Jiang, Xingzhen Lao, et al. Dramp 4.0: an open-access data repository dedicated to the clinical translation of antimicrobial peptides. *Nucleic Acids Research*, 53(D1):D403–D410, 2025.

[5] Nelson G. Oliveira Júnior, Camila M. Souza, Danieli F. Buccini, Marlon H. Cardoso, and Octávio L. Franco. Antimicrobial peptides: Structure, functions and translational applications. *Nature Reviews Microbiology*, 23(11):687–700, 2025.

[6] Malak Pirtskhalava, Anthony A Amstrong, Maia Grigolava, Mindia Chubinidze, Evgenia Alimbarashvili, Boris Vishnepolsky, Andrei Gabrielian, Alex Rosenthal, Darrell E Hurt, and Michael Tartakovsky. Dbaasp v3: database of antimicrobial/cytotoxic activity and structure of peptides as a resource for development of new therapeutics. *Nucleic acids research*, 49(D1):D288–D297, 2021.

[7] Anand Singh Rathore, Shubham Choudhury, Akanksha Arora, Purva Tijare, and Gajendra P.S. Raghava. Toxinpred 3.0: An improved method for predicting the toxicity of peptides. *Computers in Biology and Medicine*, 179:108926, 2024.

[8] Johan Svenson, Natalia Molchanova, and Christina I. Schroeder. Antimicrobial peptide mimics for clinical use: Does size matter? *Frontiers in Immunology*, 13:915368, 2022.

[9] Christina Wang, Sam Garlick, and Mire Zloh. Deep learning for novel antimicrobial peptide design. *Biomolecules*, 11(3), 2021.

[10] Jacob Witten and Zack Witten. Deep learning regression model for antimicrobial peptide design. *BioRxiv*, page 692681, 2019.

[11] Zhenyu Yang, Xiaoxi Zeng, Yi Zhao, and Runsheng Chen. Alphafold2 and its applications in the fields of biology and medicine. *Signal Transduction and Targeted Therapy*, 8(1):115, 2023.
